# Learning From the European Experience of Using Targets to Improve Population Health

**Published:** 2010-08-15

**Authors:** Peter C. Smith, Reinhard Busse

**Affiliations:** Imperial College Business School and Institute for Global Health; Berlin University of Technology, Berlin, Germany

## Abstract

Health targets have become a widely used instrument to promote population health. We describe the experience in England, where the use of targets has reached the most advanced stage of development, and other European countries. The experience demonstrates that targets may change the behavior of a health system, probably to a larger extent than many other policy instruments, if incentives are aligned correctly and if measures to deal with unintended effects are put in place.

## Introduction

Health targets are a tool designed to improve health and health system performance. They have been widely used in Europe, and governments that use them express a commitment to achieving specified results in a defined time and monitoring progress toward broader goals and objectives. Targets may be quantitative (eg, an increase of the vaccination rate by X%) or qualitative (eg, the introduction of a national screening program), and they may be based on health outcomes (eg, reduction in deaths) or processes (eg, screening activity). The introduction of the concept into the health sector is often traced to the publication of the World Health Organization's *Health for All* strategy in 1981 (1).

A large body of literature reflects the growing and sustained interest of governments in health targets and their role in the health system (2). This literature distinguishes aspirational, managerial, and technical targets, ranked in terms of the extent to which they prescribe what should be achieved and how (3). We discuss the experience in Europe with health targets as a means of promoting population health, with a particular focus on England where the use of targets has reached the most advanced stage of development (4).

## Targets in the English Health System

The first concerted attempt to introduce targets into English public health was the *Health of the Nation* strategy, launched in 1992 (5). The intent was to encourage local health authorities to focus on securing good health for their population. Initially, 5 key areas were selected for action: coronary heart disease and stroke, cancer, mental illness, HIV/AIDS and sexual health, and accidents.

In 1998, an independent evaluation of *Health of the Nation* concluded that its "impact on policy documents peaked as early as 1993; and, by 1997, its impact on local policy-making was negligible" (6). Health authorities thought they had more pressing concerns than public health, and therefore concentrated on operational issues such as reducing waiting times and securing budgetary control.

When Tony Blair became prime minister in 1997, his government was committed to evidence-based policy, systematic priority setting, and explicit performance targets throughout public services. In 1998, his government implemented a series of public service agreements (PSAs) with each ministry to signal priorities for all government activity. These priorities were a series of specific objectives, expressed as a measurable target, and were expected to be achieved in a designated time.

A distinctive feature of PSAs was the intent to focus on the outcomes of the public services, rather than operational activities. The PSA process signaled the government's determination to make the management of public services more transparent and to give departments clear statements of priorities. To illustrate the issues, we use the 2004 PSA targets in health and health care, which were based on 4 broad objectives (7): 1) improve the health of the population, that is, increase life expectancy at birth to 78.6 years for men and to 82.5 years for women by 2010; 2) improve health outcomes for people with long-term conditions; 3) improve access to services, in particular waiting times; and 4) improve the patient and user experience.

A central role of the health ministry was to devise operational instruments that transmit these national PSA targets to the local level. The most important initiative was developing a system of "performance ratings" for individual National Health Service organizations. Every organization was ranked annually on a 4-point scale (0-3 stars) according to a series of approximately 40 performance indicators intended to reflect the objectives of the National Health Service, as embodied in the PSA targets (8).

Performance ratings have improved some aspects of health services (9). For example, long waits for nonurgent inpatient treatment were rapidly eliminated. Moreover, targeted aspects of English health care have improved markedly compared with health care in Wales and Scotland, which have no PSAs or performance ratings (10).

It has proved less straightforward, however, to establish effective local targets from objectives such as reductions in deaths from heart disease and cancer, reductions of health inequalities, and reductions in rates of smoking, childhood obesity, and teenage pregnancy. Local managers have concentrated on readily managed aspects of health care, and public health has not received the sustained managerial attention given to the targets for health service delivery (11).

The PSA system has nevertheless led to sustained monitoring of the chosen population health targets and health disparities, and the ministry is held accountable for performance. The health inequalities targets have been regularly monitored by an external advisory group, but it is not clear why and how the targets were chosen, whether the observed improvements are attributable to the efforts of the health ministry, and what action should be taken when the measured performance indicated a possible failure to achieve a target (12).

A parallel initiative has been the development of a quality and outcomes framework (QOF) incentives scheme for primary care physicians (general practitioners). The QOF is one of the most ambitious attempts yet to combine clinical quality targets and incentives into physician remuneration (13). It emphasizes clinical prevention, and the earnings of individual practitioners are at risk if they do not meet quality goals. The intention is that the primary care interventions it encourages, such as smoking cessation advice, blood pressure and cholesterol control, and regular monitoring of chronic disease, will lead to a healthier population and will reduce future health care expenditures. Regrettably, researchers have been hampered in efforts to evaluate its success in improving health by the lack of reliable baseline data against which to measure improvements in health attributable to the QOF (14).

Targets have certainly delivered noteworthy successes in England, such as the more equitable management of coronary heart disease across ethnic groups (15). However, alongside the improvements in many of the measured targets are widespread reports of adverse side effects (16). Examples include neglect of unmeasured aspects of performance (eg, clinical priorities being sacrificed in the pursuit of reduced waiting times), distorted behavior (such as refusing to admit patients to accident departments until a 4-hour waiting time target was achievable), and fraud. Unintended and adverse responses such as these were predictable. They reflect the potential power of targets in affecting behavior but also emphasize the need to consider the incentives inherent in any targets regime and the need to use counteracting instruments where necessary (17).

## Discussing the Lessons

Drawing on the experience of England and case studies from other countries (2), we discuss 6 lessons that arise from the use of population health targets.

### Who should choose the targets?

In principle, it seems laudable for an elected government to set out its objectives and targets in an explicit fashion. Targets serve many purposes, but one is to enhance the accountability of government to parliament and the electorate. Lack of an adequate accountability framework may lead to the failure of target setting to achieve its objectives. For example, in Hungary, where accountability arrangements were not aligned with the public health focus of targets (18), achievement was monitored at the national level, but no mechanism secured the commitment of organizations and practitioners capable of influencing outcomes.

The English process succeeded in that much of the public debate surrounding targets referred less to the principle of setting targets and more to the details of what those targets should be. Disagreement remains about the processes by which priorities are chosen and targets set. For example, there is an argument that the health service professionals should have more say in influencing the nature of targets, when outcomes rely so heavily on their engagement and commitment. However, the priorities and working practices of those professionals may impede progress toward better performance. To some extent, outcome-related targets seek to challenge traditional ways of delivering services and will, therefore, at times come into conflict with the professions.

Some commentators argue that service users should have more say in setting targets. Wide consultation with user groups can identify priorities for improvement. However, particularly in population health, setting objectives involves considerations beyond the immediate beneficiaries of a particular service, such as the taxpayer perspective, the interests of future users, and the interests of users of other services. The user perspective cannot be the sole influence on priority setting.

Consensus and ownership have nevertheless been seen as imperative to elicit acceptance of country-based targets. In Catalonia, health councils were created at the central and provincial levels to encourage citizens' groups to take an active part in target setting (19). In Flanders, local health networks were established to encourage the exchange of information between local organizations and offer a focal point for preventive actions (20). France established national and regional health conferences that allowed stakeholders the opportunity to debate existing health problems and foster partnerships (21).

Any government seeking to implement population health targets should reach consensus concerning the choice of objectives and the nature of the targets by consulting with relevant stakeholders. However, uncritical accommodation of every interest group would render the target process meaningless; for example, it could lead to an unwieldy proliferation of priorities. A prime role of government is to balance conflicting claims on public resources, and targets should, in the end, be an explicit and succinct statement of the government's choice in that respect.

### How many targets should be chosen?

Multiple objectives are an inescapable characteristic of health services. However, one of the intentions of any targets regime is to focus on a limited number of objectives. Many schemes have failed to recognize this, for example in Italy (100 targets) and Andalucía (84 targets) (22). In England, after some early failures, later PSAs focused on a reduced number of targets.

If a domain is not included in the targets regime, this is not necessarily an indication that it is unimportant. Rather, the key focus of targets should be where change is required, and maintenance of standards in other domains should be secured through other instruments, such as routine regulation, inspection, or market mechanisms.

### When should outcomes be used as a basis for targets?

In principle, a focus on outcomes should enable health care providers to look beyond traditional organizational boundaries and ways of delivering their services. However, some outcomes are intrinsically difficult to measure. Even if they can be measured, outcomes such as reduced deaths from smoking can take years to materialize, beyond the lifetime of most governments. Furthermore, many public health outcomes are particularly vulnerable to influences beyond the control of health agencies. Each of these difficulties offers those agencies an excuse for apparent failure and can undermine the targets process.

Conversely, the use of process measures can distort behavior and lead to unintended effects. For example, the QOF "smoking cessation" target may have led to an undue emphasis on delivering advisory consultations without any attention to outcomes in the form of sustained cessation. If such process targets are used, additional assurance may be needed to ensure that the desired outcomes have been secured. Although outcome measures address what matters and are less vulnerable to distortion, there will be occasions when a carefully chosen process measure — one that evidence shows is clearly linked to the eventual outcome — may form a more effective basis for a target.

### How should targets be quantified?

Once objectives have been identified, a central feature of the debate becomes how the associated targets should be set, in terms of the measurement instrument to be used and the level of attainment to be required. The literature suggests that targets should be SMART — specific, measurable, achievable, realistic, and timed (3). The Royal Statistical Society (23) presents a set of desirable general principles for setting targets, which include following:

1. Indicators should be directly relevant to the primary objective or be an obviously adequate proxy measure.
2. Definitions need to be precise, practicable, and consistent over time.
3. Indicators should be straightforward to interpret and avoid perverse incentives.
4. Indicators should be based on adequate sample sizes, and technical properties of the indicator should be satisfactory.
5. Indicators should not impose an undue burden in terms of cost, personnel, or intrusion on those providing the information.

In practice, few targets regimes have adhered to principles such as these. For example, Swedish public health targets were not explicit enough to act as a lever for operational action (24). Some targets might be little more than unattainable aspirations, while others can be secured with little effort on the part of ministries. Furthermore, conflicting pressures exist in any targets regime. To be effective managerial instruments, targets should be stretching but attainable, suggesting (for example) a 1 in 3 risk of failure. However, few governments would want to be confronted with such a high proportion of failures. From an accountability perspective, a government would wish to think that all targets could be attained.

This scenario occurred in the Netherlands during the early 1990s, where the secretary of state for health avoided using quantitative health targets because of the political accountability those targets would create (3). Similarly, Russia has experienced politically driven target setting, where the targets set were neither relevant nor necessary. Health was seldom a priority on the policy agenda in the Union of Soviet Socialist Republics or subsequently in the Russian Federation, and generally, when targets were set they were broadly defined, infrastructure-oriented, and almost never outcome-oriented. In many cases, the targets required no change in policy to achieve them (25). It is difficult to see how this tension can be satisfactorily resolved, unless the political process becomes mature enough to recognize that some failure is inevitable and not necessarily adverse if progress is being secured.

### How should cross-ministerial targets be handled?

Given the many determinants of health, involving actions by organizations in various sectors, effective coordination among responsible actors has emerged as a key issue. In particular, a focus on health outcomes sometimes gives rise to strategies that are not obviously attached to a particular ministry, leading to the need to specify "joint" targets that transcend departmental boundaries. These are particularly important in the public health domain. An assessment of the English childhood obesity PSA target found no ready solutions but advocated much stronger collaboration between national and local government and stronger engagement with nongovernmental organizations (26). Cross-sectoral targets give rise to problems of coordination, persuasion, and engagement that must be addressed if they are to be successful.

Where this coordination takes place will depend on the governance structures already in place and the forums in which key actors can meet. This may be easier where responsibility for health lies in local or regional government, as in Scandinavia. Other countries have faced a different challenge with intersectoral targets. Although they have stressed the need to involve the many sectors whose actions contribute to health, they have often not included the health care sector itself. By not including that sector, health targets become a peripheral issue, thereby diluting the potential effect of that sector (27).

### How should national objectives be transmitted to local organizations?

Attainment of national targets usually relies on improvement in local organizations charged with delivering services. It would, however, be inappropriate to set the same targets for every locality regardless of its existing level of attainment and the difficulty of the local circumstances. Organizations already performing well would have no incentive to improve, whilst those with disadvantaged populations might stand no chance of success and become alienated. If such regimes were sustained, it may become difficult to recruit key managers and professionals in disadvantaged areas, exacerbating existing problems. As a result, many countries have introduced more subtle targets regimes for local organizations, seeking to encourage all organizations to improve in the chosen measures, from whatever baseline they start.

The tension between national objectives and local discretion has become an unresolved issue in targets regimes. In England, the "must do" nature of local health targets put pressure on some local organizations, precluding any serious consideration of separate local priorities. The prevailing lack of flexibility was highlighted in a report by the Audit Commission (28) that criticized the neglect of local government discretion in earlier PSA targets. There is now increased interest in England on public reporting of local levels of attainment, regardless of which agency is nominally accountable (29). In short, targets programs have often been disseminated in a top-down manner with little effort to ensure involvement of key actors at the grassroots level (27). For the future, a sense of ownership and accountability needs to be developed among those who implement health targets.

## Conclusion

Health targets have become a widely used instrument to promote population health. The lessons we have described demonstrate that targets may secure a real change in the behavior of a health system, probably to a larger extent than many other policy instruments, if incentives are aligned correctly and if measures to deal with unintended effects are put in place.
